# Data on the effect of the dispersion of functionalized nanoparticles TiO_2_ with photocatalytic activity in LDPE

**DOI:** 10.1016/j.dib.2017.12.032

**Published:** 2017-12-16

**Authors:** Alvarado Jahell, Acosta Guillermo, Perez Fatima

**Affiliations:** a Nanotechnology Incubator I2T2, Apodaca, Nuevo León 66629, Mexico; b Nanomateriales SA de CV, San Pedro Garza Garcia, Nuevo Leon 66269, Mexico; c CONACYT-IPICYT/División de Ciencias Ambientales, 78216, Mexico

## Abstract

This article contains the dataset referring to the article "Study of the effect of the dispersion of functionalized nanoparticles TiO_2_ with photocatalytic activity in LDPE" (Jahell et al., 2016) [1]. It includes the FT-IR data of the functionalized nanoparticles of TiO_2_ with Hexadecyltrimethoxysilane in different degrees of functionalization, thermogravimetric analysis, distribution and particle size in the polymer matrix by scanning electron microscopy (SEM), carbonyl index, gravimetry and scanning electron microscopy of the nanocomposite degraded by UV radiation.

**Specifications Table**

**Table:** 

Subject area	*Chemistry*
More specific subject area	*Polymers*
Type of data	*Figures*
How data was acquired	*FT-IR-Affinity Shimadzu, TGA-50 Shimadzu, Scanning Electron Microscope (SEM, STEM, NANOSEM 200-FEI)*
Data format	*Analyzed*
Experimental factors	*The nanoparticles were recovered by filtration and washed with ethanol to remove the coupling agent unreacted. the particles were dried in a vacuum oven at 80 °C for 12 h*.
Experimental features	*FT-IR was obtained by ATR and the TGA at a heating rate of 10 °C/min in air*
Data source location	*Monterrey, Nuevo Leon, Mexico*
Data accessibility	*Data is provided in the article*

**Value of the data**

• This work shows how the degree of functionalization is relevant to determine the distribution in a polymer matrix and how this affects the process of photocatalytic degradation.
• The data presented corroborate the different degrees of functionalization of nanoparticles used for the formation of nanocomposites.
• The data presented show degradation on the surface of a polymer in a photocatalytic process.

## 1 Data

**Fig. 1:**
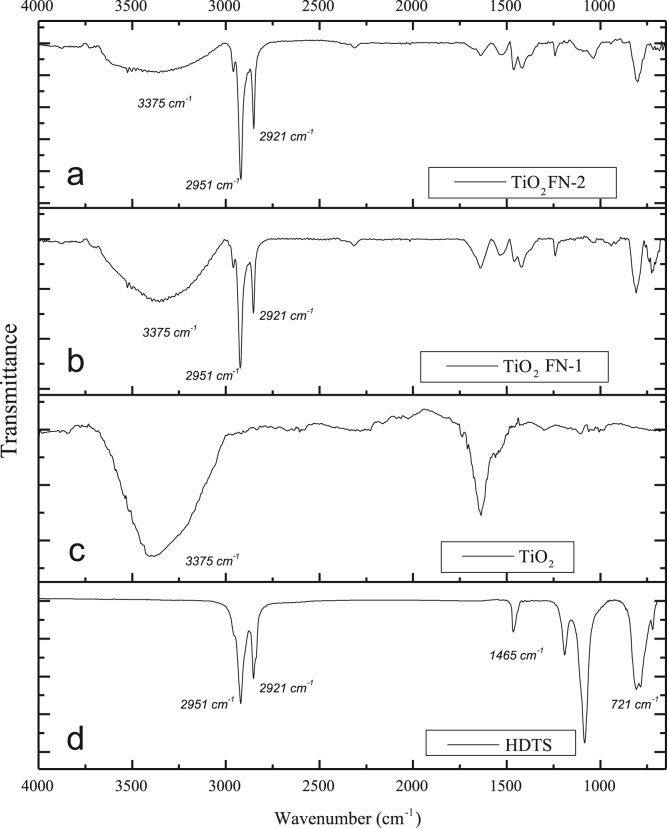
FT-IR Titanium dioxide nanoparticles (TiO_2_), Hexadecyltrimethoxysilane (HDTS), functionalized nanoparticles.

**Fig. 2:**
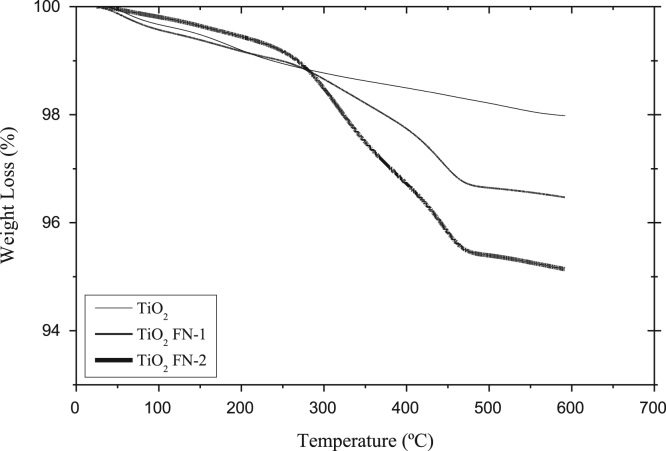
Thermogravimetric Analysis. Titanium dioxide unfunctionalized (TiO_2_), titanium dioxide functionalized (TiO_2_FN-1 and TiO_2_FN-2).

**Fig. 3:**
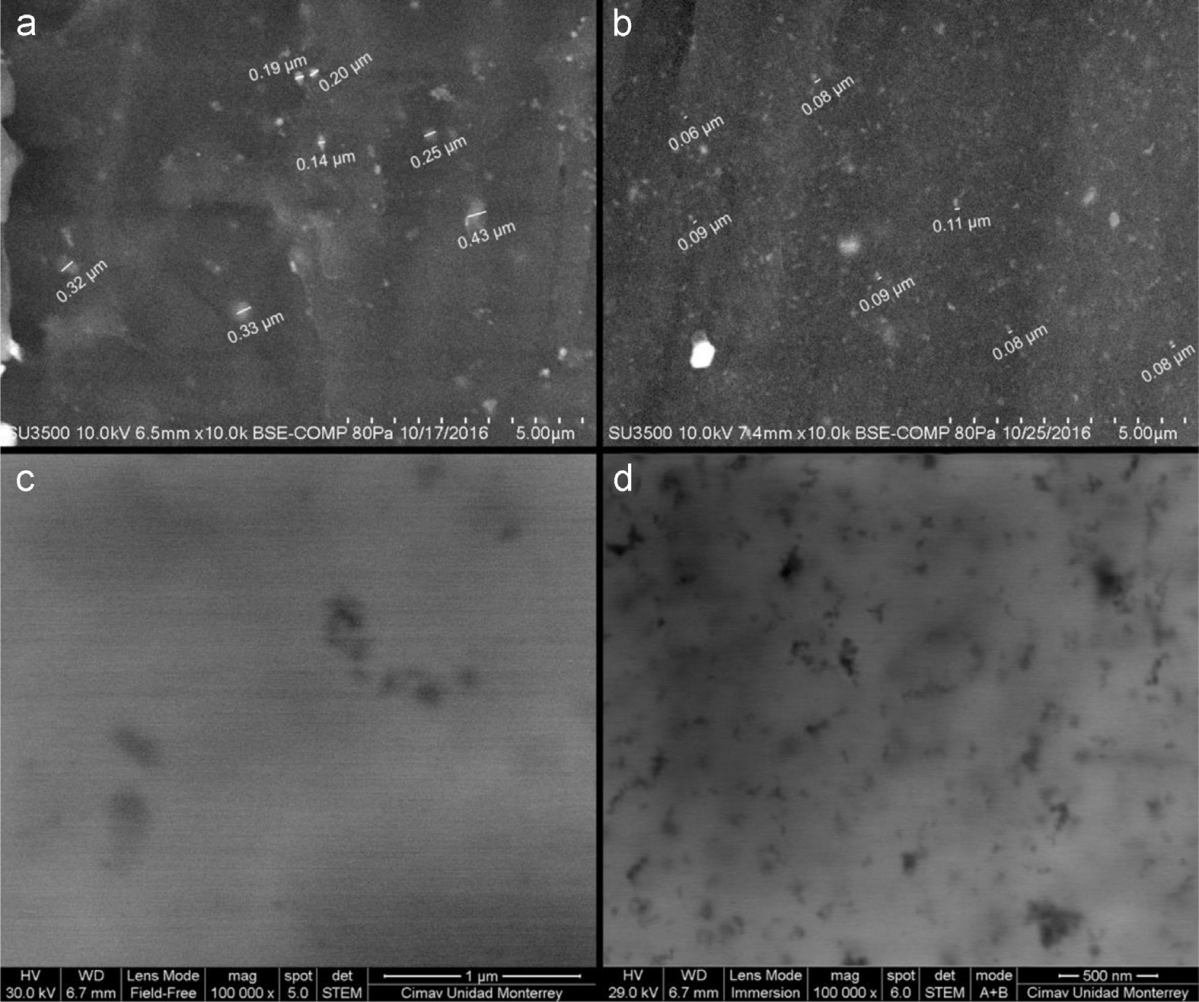
Nanocomposite LDPE-TiO_2_ (a-SEM, c-STEM), Nanocomposite LDPE-TiO_2_FN-2 (b-SEM, d-STEM).

**Fig. 4:**
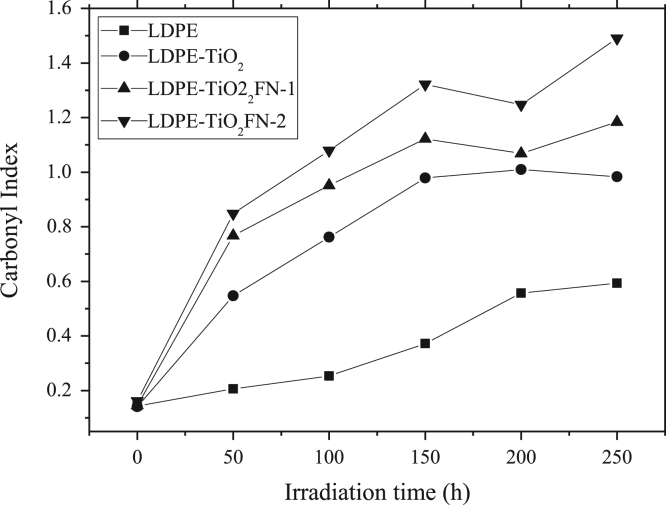
FT-IR Analysis-Carbonyl Index.

**Fig. 5:**
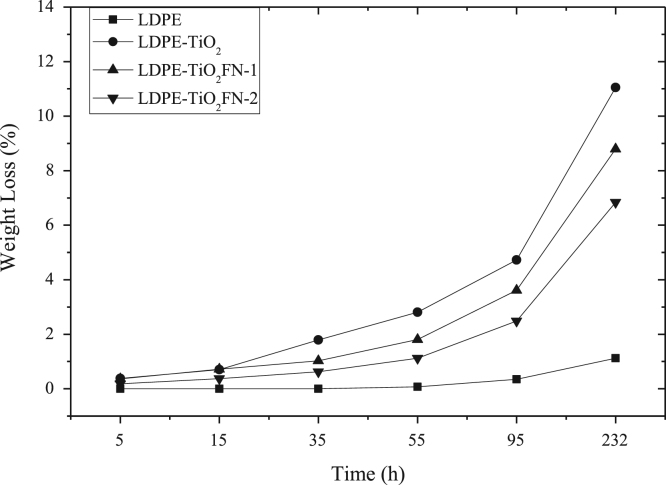
Gravimetry. LDPE (■), TiO_2_ wt. (•), TiO_2_ FN-1 (▲), TiO_2_ FN-2 (▼).

**Fig. 6:**
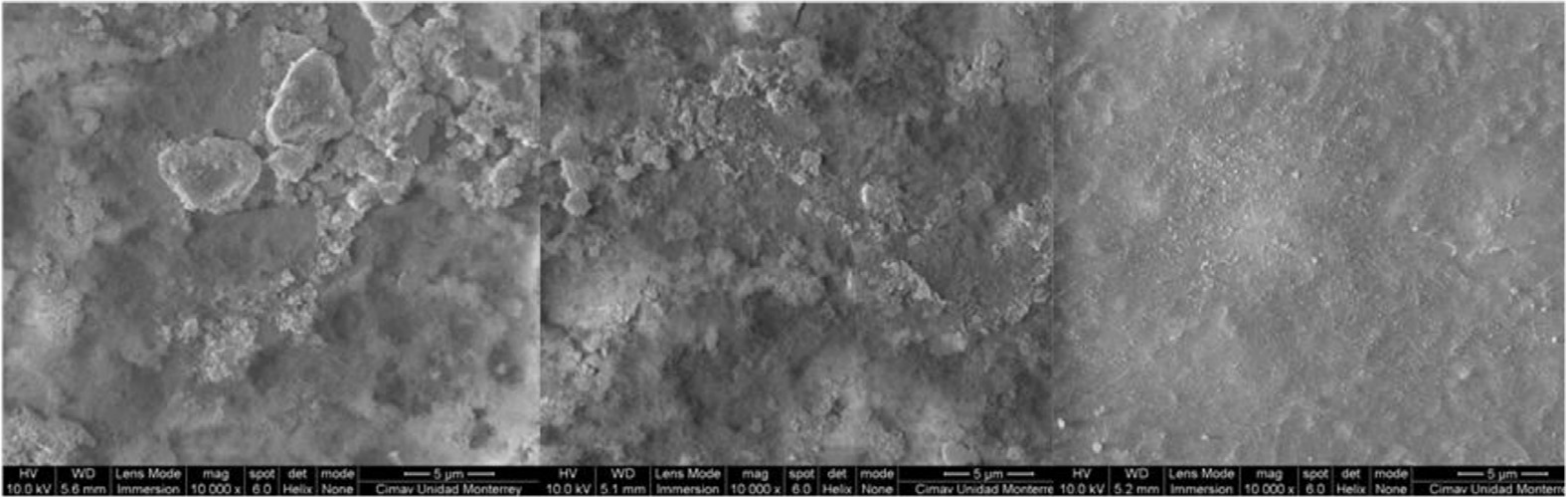
SEM- TiO_2_ (left), TiO_2_ FN-1. (middle), TiO_2_ FN -2 (right).

The data presented here include the FT-IR spectra of functionalized and unfunctionalized nanoparticles, Fig. 1. Thermograms (TGA) of the functionalized and unfunctionalized particles, Fig. 2. The images of the nanocomposites by the STEM technique, Fig. 3. The carbonyl content of the nanocomposites degraded and analyzed by FT-IR, Fig. 4. The data of the gravimetric analysis of the nanocomposites at different times of exposure to UV radiation, Fig. 5 and scanning electron microscopy images of the Degraded nanocomposites Fig. 6 [1].

## 2 Experimental design, materials and methods

The functionalization process was performed using an adaptation of the method used by Nguyen et al. [2] in which a suitable amount of TiO2 nanoparticles was added into ethanol solution; the dispersion was subjected to sonication for 3 (10 min) cycles with a 5 min rest. Subsequently, the dispersion was stirred to achieve a temperature of 65 °C. Once the desired temperature was reached, the coupling agent (HDTS) was dosed drop-by-drop according to the desired degree of functionalization (Table 1). The reaction temperature increased to 78 °C and refluxed for 3 h. The nanoparticles were recovered by filtration and washed with ethanol to remove the unreacted coupling agent. Finally, the particles were dried in a vacuum oven at 80 °C for 12 h. Table 1 includes the experimental data used for the functionalization of the nanoparticles with their respective nomenclature.

**Table 1:** Composition of the reaction medium for functionalization.

Sample ID	*EtOH (wt%)*	*TiO_2_ (wt%)*	*HDTS (wt%)*
TiO_2_	99	1	0
TiO_2_ FN-1	98.9	1	0.1
TiO_2_ FN-2	98.5	1	0.5

Infrared spectroscopy (FT-IR-Affinity Shimadzu) was used to determine the degree of functionalization; the different samples were subjected to a washing process and then exposed to a beam of infrared light.

Thermogravimetric analysis was performed to determine weight loss using TGA-50 Shimadzu, at a heating rate of 10 °C/min in air. Functionalizing agent content was determined by the following Eq. [3]:

$$n_{f}=10^{6}\frac{∆m_{s}}{m_{f}S_{s}{\mathrm{MW}}_{\mathrm{silane}}}$$

where n_f_ is functionalizing agent content (µmol/m^2^), Δm_s_ is HDTS weight gain for the TiO_2_ (g) and measurement in TGA, *m*_s_ is the mass of the TiO_2_ (g), *S*_s_ is the specific area of the TiO_2_ (m^2^/g), and MW_silane_ is the molecular weight of the bonded silane molecule (g/mol). In this work the molecular weight of HDTS is 325 g/mol considering a monodentate bond at the particle surface.

The preparation of nanocomposites consists of mixing polymer pellets at a concentration 3% by the weight of the nanoparticles; three samples were processed: one with unfunctionalized nanoparticles, and two with functionalized nanoparticles at a concentration of TiO_2_ FN-1 and TiO_2_ FN-2 of coupling agent, respectively. The mixing process was carried out in a turbomixer at 1000 rpm for 2 min to prevent overheating. After mixing, the different samples were extruded in a co-rotating twin screw extruder with two intensive areas of mixed brand Rondol (*L*/*D* = 25:1) to obtain pellets. The extrusion process was performed employing the following temperature profile: 145; 185; 185; 195 and 200 °C. The pellets were processed in a hydraulic hot-press (Carver Press-8 t, 190 °C) to obtain nanocomposite films of 0.4 mm thickness. The concentration of nanoparticles in the polymer utilized in this study was determined considering that Daneshpayeh and collaborators in 2016 conducted an optimized concentration of TiO_2_ in a polypropylene matrix to improve the mechanical properties, where the highest tensile strength is obtained at a concentration of 3% by weight [4].
