# Supplementary material for: Data on the effect of the dispersion of functionalized nanoparticles TiO2 with photocatalytic activity in LDPE
Source: Data Brief. 2017 Dec 16;16:1038–43. doi: 10.1016/j.dib.2017.12.032 (PMC5752085; doi:10.1016/j.dib.2017.12.032)
Supplement: Supplementary file 1 — Transparency document [file mmc1.docx]

**Conflicts of Interest Statement**

Manuscript title: *Data on the effect of the dispersion of functionalized nanoparticles TiO_2_ with photocatalytic activity in LDPE*

The authors whose names are listed immediately below certify that they have NONE declarations of interest.

Author names:

- Alvarado Jahell
- Acosta Guillermo
- Perez Fatima
